# Synthesis and Characterization of Types A and B Gelatin Methacryloyl for Bioink Applications

**DOI:** 10.3390/ma9100797

**Published:** 2016-09-24

**Authors:** Bae Hoon Lee, Nathaniel Lum, Li Yuan Seow, Pei Qi Lim, Lay Poh Tan

**Affiliations:** 1 School of Materials Science and Engineering, Nanyang Technological University, 50 Nanyang Avenue, Singapore 639798, Singapore; NLUM001@e.ntu.edu.sg (N.L.); M130073@e.ntu.edu.sg (L.Y.S.); PLIM023@e.ntu.edu.sg (P.Q.L.); 2 Singapore Centre for 3D Printing (SC3DP), Singapore 639798, Singapore

**Keywords:** gelatin, methacryloyl, bioink, type A and type B, 3D bioprinting

## Abstract

Gelatin methacryloyl (GelMA) has been increasingly considered as an important bioink material due to its tailorable mechanical properties, good biocompatibility, and ability to be photopolymerized in situ as well as printability. GelMA can be classified into two types: type A GelMA (a product from acid treatment) and type B GelMA (a product from alkali treatment). In current literature, there is little research on the comparison of type A GelMA and type B GelMA in terms of synthesis, rheological properties, and printability for bioink applications. Here, we report the synthesis, rheological properties, and printability of types A and B GelMA. Types A and B GelMA samples with different degrees of substitution (DS) were prepared in a controllable manner by a time-lapse loading method of methacrylic anhydride (MAA) and different feed ratios of MAA to gelatin. Type B GelMA tended to have a slightly higher DS compared to type A GelMA, especially in a lower feed ratio of MAA to gelatin. All the type A and type B GelMA solutions with different DS exhibited shear thinning behaviours at 37 °C. However, only GelMA with a high DS had an easy-to-extrude feature at room temperature. The cell-laden printed constructs of types A and B GelMA at 20% *w*/*v* showed around 75% cell viability.

## 1. Introduction

Three-dimensional (3D) printing is the new cutting edge technology that has been developed rapidly for various applications such as bioprinting [1,2,3]. As 3D printing technology evolves, an increasing number of materials such as acrylonitrile butadiene styrene, photo-curable resins, and even stainless steel have been developed and used along for manufacturing all forms of complex delicate building blocks. This development of 3D printing has gained much attention from various industries because of its ability to fabricate high-precision products and reduce the product design cycle for various industries [4,5]. 3D bioprinting is another powerful tool from 3D printing technology for tissue engineering and regenerative medicine applications [6,7,8,9]. A common method for bioprinting is through an extrusion technique, where a mixture of viscous cell-supportive materials and biologically active components including cells is dispensed onto a substrate [10,11,12,13,14].

3D bioprinting is basically incorporating a 3D printing technique into tissue engineering application. Currently, there are numerous studies that focus on 3D bioprinting of cells and scaffold materials together to create 3D artificial constructs for organ transplantation and drug screening [15,16]. 3D bioprinting is not only useful for 3D cell interaction studies, but also it potentially helps in regeneration therapy. By using regeneration therapy, an organ can be regenerated and the chance of patients receiving an organ transplant will increase.

In parallel with extensive exploration in the field of 3D bioprinting, the development of a suitable bioink is another important research area needed to meet all requirements for printing different types of artificial tissues and organs [17,18,19]. Basically, a mixture of materials and cells is known as a bioink in the 3D bioprinting industry. An ink material can become a scaffold after a certain treatment such as heat, ions, enzymes and light. Examples of typical bioink materials are alginate [20], gelatin [21], and gelatin methacryloyl [16,22,23]. Ideal bioinks have some important characteristics such as low cost, printability, cell compatibility and cell-specific functionality [10]. A bioink should allow cell encapsulation, should be printable with a good resolution, and should ideally guide cells in the construct towards specific functions or behaviour.

Gelatin methacryloyl (GelMA) is an attractive photocurable material which is made from chemically modified gelatin with methacrylic anhydride (MAA) [24]. GelMA synthesis was first introduced by Van Den Bulcke et al. [25]. Recently, we developed an efficient and controllable synthesis of type A GelMA for mechanically stiff hydrogels using a sequential time-lapse loading method of MAA as well as one-pot GelMA synthesis [26,27]. GelMA is able to form a stable chemical gel with a water soluble photoinitiator upon exposure to a long wavelength ultraviolet (UV). The resultant hydrogel retains its physical form in body temperature and maintains its structural stability. This is in contrast to pure gelatin which is soluble at body temperature [25]. GelMA has controllable mechanical properties depending on its degree of methacryloylation and its concentration. In addition, GelMA retains its intrinsic biological properties such as enzyme-degradability and cell-adhesion promotion [23,24,28]. In these respects, GelMA could be a good candidate for bioink applications. The research conducted by Billiet et al. in 2014 showed that the 3D printed GelMA scaffold had excellent cell viability and scaffold stability for 14 days, indicating that GelMA could have great potential as a bioink for 3D bioprinting [16]. Most of the existing research studies carried out on GelMA focused on rheological studies [21], printing parameters [16] and cell viability [16,21,22].

However, there has been little research on comparing type A GelMA with type B GelMA as to synthesis, rheological properties, and printability for bioink applications. In this report, we synthesized type A and type B GelMA with different degrees of substitution, and measured their rheological properties, and discussed feasibility of type A and type B GelMA as a bioink material.

## 2. Experimental Section

### 2.1. Types A and B GelMA Synthesis

Gelatin Methacryloyl (GelMA) was synthesized in a buffer (0.1 M CB) of sodium carbonate (3.18 g) and sodium bicarbonate (5.86 g) in 1 L of distilled water. Gelatin (20 g) was added to 200 mL of the CB solution. Two types of gelatin were used-type A (Bloom 175 g, porcine skin, Sigma-Aldrich, St. Louis, MO, USA) and type B (Bloom 225 g, bovine skin, Sigma-Aldrich, St. Louis, MO, USA). The gelatin solutions were adjusted to pH 9 by addition of 5 N NaOH to promote the reaction of the amino groups on gelatin. Methacrylate anhydride (MAA, 94%, Sigma-Aldrich, St. Louis, MO, USA) was added to the gelatin solutions at various feed ratios of 0.1:1, 0.05:1, 0.025:1, and 0.0125:1 (mL of MAA to g of gelatin), and the addition of MAA was divided into 6 separate times with an average of 11 min between each addition. As the reaction proceeded, the pH decreased. As such, the pH of the reaction solutions was adjusted to pH 9 after each addition for optimal reaction of gelatin with MAA. After around 1.5 h of the reaction, 1 N HCl was added to the solutions to be adjusted to the physiological pH of 7.4. The solutions were then filtered with 70 mm paper filter, followed by an antibacterial filter (0.2 μm). Then, the solutions were dialyzed for about 7 h through Minimate™ Tangential Flow Filtration system (Pall Corporation, Port Washington, WA, USA) which filtered out small molecules of less than 10 kDa, unreacted MAA, and methacrylic acid byproduct. After dialysis, the solutions were placed at −70 °C for 3–5 h, and finally lyophilized to obtain the dry GelMA products.

### 2.2. Determination of GelMA DS

To determine the degree of substitution, TNBS (2,4,6-Trinitrobenzenesulfonic acid, Sigma-Aldrich, St. Louis, MO, USA) method was used. GelMA and pure gelatin (type A and type B) were diluted in 0.1 M sodium bicarbonate buffer to 1.6 mg·mL^−1^. Then, 500 μL of 0.2% TNBS solution was added to 500 μL of each sample and placed in 37 °C for 3 h. Subsequently, 250 μL of 1 M hydrochloric acid and 500 μL of 10% *w*/*v* sodium dodecyl sulphate were added to stop the reaction. The absorbance of the samples was then measured at 335 nm. The molar concentration of free primary amino groups was compared to glycine standard solutions at concentrations of 0, 0.8, 8, 16, 32, and 64 μg·mL^−1^.

In order to verify the methacryloylation, ^1^H NMR measurement was conducted. Types A and B GelMA samples were separately dissolved at around 50 mg·mL^−1^ in deuterium oxide (Merck, Kenilworth, NJ, USA) at 37 °C, and the 1H chemical shifts of each sample were observed.

### 2.3. Viscoelastic Properties of Types A and B GelMA

Characterization of the mechanical properties of the GelMA hydrogels was carried out via Rheometer (Anton Paar Physica, Ostfildern, Germany) MCR 501 testing using an Anton Paar CP25/TG 25-mm diameter cone-plate geometry with one-degree angle. The GelMA was photo-crosslinked in situ, and time sweep and frequency sweep tests were performed. The parameters for the time sweep test were set at frequency of 1 Hz for the entire duration of the test, and shear strain of 2% for about 6 min. The samples were irradiated with UV for 60 s after the time sweep test started. For the frequency sweep test of the cured GelMA, the parameters were set at 0.5% shear strain within the linear viscoelastic region and the frequency range of 0.1 to 10 Hz. About 70 μL of GelMA solutions (10%, 20%, and 30% *w*/*v*) containing 0.1% Irgacure 2959 (2-Hydroxy-4′-(2-hydroxyethoxy)-2-methylpropiophenone, Sigma-Aldrich, St. Louis, MO, USA) was used for each test and the temperature was set at 37 °C.

To investigate the effect of shear rate on shear stress and viscosity of GelMA at 37 °C and 25 °C, a rheology test was conducted under shear rate from 1 to 1000 s^−1^. For testing the effect of temperature on the viscosity of GelMA, the shear rate was kept constant at 50 s^−1^ and the viscosity of the GelMA samples was measured from 40 °C to 20 °C at the speed of 2 °C·min^−1^. The storage and loss modulus profiles of the GelMA samples were obtained at 0.5% shear strain and a frequency range of 0.1 to 10 Hz, and both at 37 °C and 25 °C.

### 2.4. Printability of Types A and B GelMA

Type A GelMA 2.2 and type B GelMA 2.2 with 20% *w*/*v* were tested for printability in Desktop Robot 2300N (SAN-EI-TECH Ltd., Chiba, Japan). Both samples were transferred and left to cool to room temperature (25 °C) in syringe before printing. The extrusion pressure was varied from 0.1 to 0.4 MPa in order to determine an optimal pressure for obtaining a good resolution print for both types A and B GelMA solutions. A conical needle size with gauge size 25 (inner diameter = 0.25 mm) was used. The printing temperature was kept at room temperature (25 °C) and printing speed used in the setup is 10 mm·s^−1^. A pressure of 0.15 MPa was finally selected for type A GelMA and type B GelMA printing. For actual printing, a 3D grid pattern with the dimension of 2 cm × 2 cm with line spacing of 4 mm was printed using both types A and B GelMA with curing or without curing.

### 2.5. 3D Printing of Cell-Laden Constructs of Types A and B GelMA

Human hepatocarcinoma cells (Huh-7.5, Apath) were cultured in Dulbecco’s Modified Eagle’s Medium (DMEM, Hyclone) supplemented with 10% fetal bovine serum (FBS, Hyclone) and 1% antibiotic/antimycotic (ABAM, Life Technologies, Gaithersburg, MD, USA) in a humidified atmosphere with 5% CO_2_ at 37 °C. Types A and B GelMA samples at 20% were mixed with 2 × 10^6^ cells in PBS containing 0.1% Irgacure 2959 and used for 3D printing of the 3D grid pattern with the dimension of 2 cm × 2 cm with line spacing of 4 mm. The cell-laden printed constructs with five layers were cured at 1–2 mW·cm^−2^ for 5 min. The cell viability on photopolymerized cell-laden print constructs of types A and B GelMA was measured using the LIVE/DEAD^®^ Cell Viability/Cytotoxicity kit (Life Technologies, Gaithersburg, MD, USA) 4 h after printing. In brief, 4 μM calcein-AM and 8 μM ethidium homodimer-1 (EthD-1) were prepared in media and added to the cell-laden print constructs and incubated at 37 °C for 1 h. Live cells stained by calcein-AM were imaged as green, and dead cells stained by EthD-1 were imaged as red by microscope. Live and dead cells (*n* = 5 images, the average total number of cells = 387) were counted using ImageJ.

### 2.6. Statistic Analysis

Statistical analysis was performed using the Microsoft Excel statistical analysis software package. Standard deviation was calculated and presented for each treatment group (mean ± SD). Significant difference among the experimental groups was determined by a two-tailed student’s *t*-test. *p* values < 0.05 were considered to be statistically significant.

## 3. Results

### 3.1. Type A and Type B GelMA Synthesis

**Figure 1:**
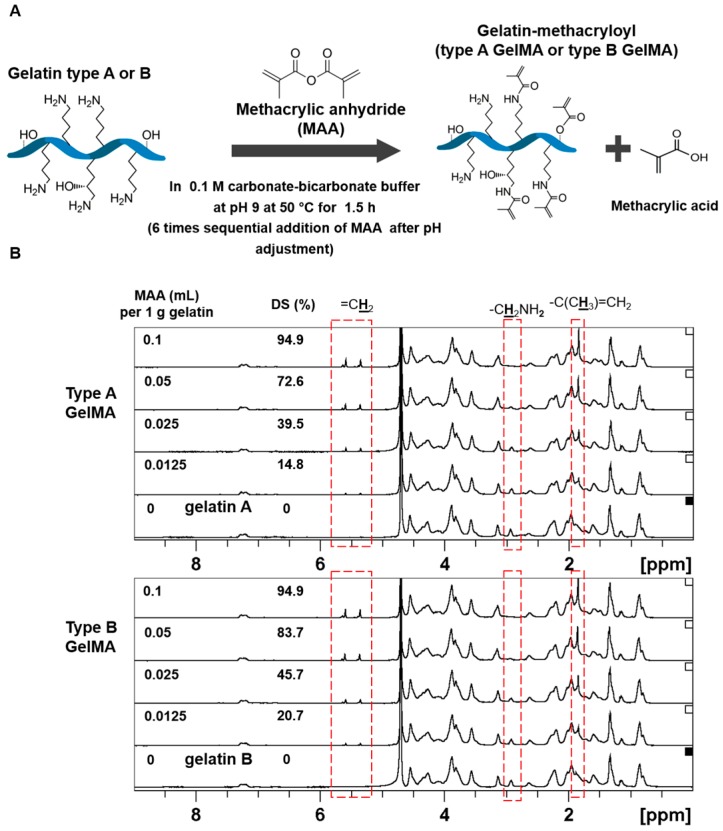
Preparation of types A and B GelMA. (**A**) Schematic illustration of GelMA reaction; (**B**) ^1^H NMR verification of types A and B GelMA. Peaks correspond to acrylic protons (2H) of methacryloylated grafts of lysine groups and those of hydroxyl lysine groups, methylene protons (2H) of unreacted lysine groups, and methyl protons (3H) of methacryloylated grafts.

**Figure 2:**
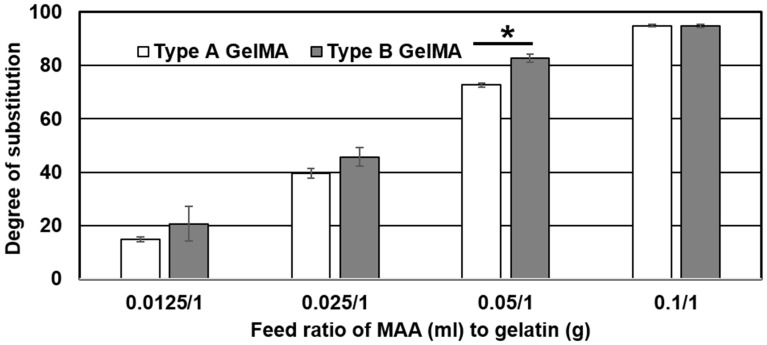
Comparison of the effect of a feed ratio of MAA/gelatin on the degree of substitution of types A and B GelMA. Error bar stands for standard deviation of means (* *p* < 0.01, *n* = 3).

Both type A and type B gelatin samples were reacted with MAA at various feed ratios in a sequential addition method similar to reported literature [26]. The degree of substitution (DS) of methacryloyl groups on both types A and B gelatin was determined by 2,4,6-Trinitrobenzenesulfonic acid (TNBS) method. Similar results and trends were found for both type A GelMA and type B GelMA as presented in Figure 1 and Figure 2. Both Types A and B GelMA showed increasing degrees of substitution with increasing feed ratios of MAA to gelatin. The increase in degree of substitution was approximately proportional to the feed ratio up to a feed ratio of MAA (0.1 mL) to gelatin (1 g). At the highest feed ratio of MAA (0.1 mL) to gelatin (1 g), the reaction reached almost complete methacryloylation of lysine groups. The degree of substitution of type B GelMA was higher than that of type A GelMA especially at a lower feed ratio of MAA to gelatin.

NMR characterization was conducted to verify that gelatin methacryloyl (GelMA) had been synthesized from the constituent gelatin and MAA. For the NMR analysis, the phenylalanine peaks (7.1–7.4 ppm) were taken as the standard among all the different samples as it was not modified by reaction with MAA. Both type A and type B GelMA samples formed new peaks from the reaction at around 5.3 and 5.5 ppm which were attributed to acrylic protons (2H) of methacryloylated grafts. The intensity of the peaks increased with the degree of methacryloylation, showing that the higher feed ratio of MAA to gelatin could produce GelMA with the higher degree of substitution. There was also a peak at around 1.8 ppm from the methyl group (3H) of methacryloylated grafts. This peak also increased with the degree of substitution, which indicates increasing methacryloylation. There was also a decreasing trend observed for the lysine methylene peak (2H) from gelatin at 2.8–3.0 ppm. This peak was the highest in pure gelatin and gradually decreased as the degree of substitution increased. As lysine is the bonding site for methacryloyl conjugation, it indicates that more lysine groups reacted with MAA and there was increased methacryloylation with lysine groups.

### 3.2. In Situ Photopolymerizing Properties of Types A and B GelMA

**Figure 3:**
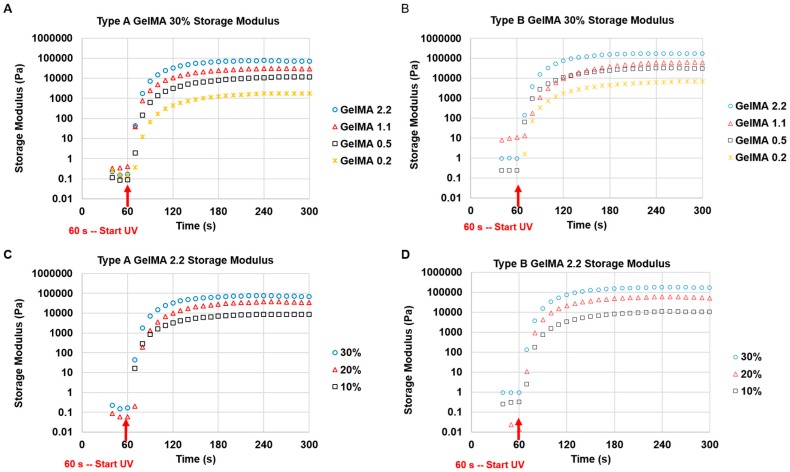
Rheological analysis of time-dependent storage modulus on UV irradiated GelMA with different DS and different concentrations at 37 °C. (**A**) 30% type A GelMA with different DS; (**B**) 30% type B GelMA with different DS; (**C**) Type A GelMA 2.2 with different concentrations (30%, 20%, and 10%); (**D**) Type B GelMA 2.2 with different concentrations (30%, 20%, and 10%).

The samples used for in situ photopolymerizing properties of GelMA were GelMA 0.2, GelMA 0.5, GelMA 1.1, and GelMA 2.2 from Type A and Type B as seen in Table S1. First, the time sweep test of the storage modulus versus time was carried out to determine the gelation behaviour during crosslinking. As presented in Figure 3, the time sweep results showed that types A and B GelMA solutions (10%, 20%, and 30%) were photocrosslinked rapidly, within 20–30 s after the exposure to UV irradiation. The storage moduli of the formed GelMA hydrogels reached a plateau 1 min after UV exposure and showed a clear trend that the storage modulus increased with an increase in degree of substitution and hydrogel concentration. For type A GelMA, GelMA 2.2 (94.9% DS), GelMA 1.1 (72.6% DS), GelMA 0.5 (39.5% DS), and GelMA 0.2 (14.8% DS) at 30% *w*/*v* exhibited 67.6, 26.7, 11.3, and 1.9 kPa, respectively. The storage moduli of GelMA 2.2 at 30%, GelMA 2.2 at 20%, GelMA 2.2 at 10% were 67.6, 34.7, and 9.1 kPa, respectively. Type B GelMA exhibited trends similar to that of type A GelMA. However, type B GelMA hydrogel showed a higher storage modulus than type A GelMA hydrogel at the same concentration. Type A GelMA 2.2 (94.9% DS) at 30% exhibited 67.6 kPa storage modulus whereas type B GelMA 2.2 (94.9% DS) at 30% exhibited 147.3 kPa storage modulus (*p* < 0.01, *n* = 3).

### 3.3. Viscoelastictic Properties of Types A and B GelMA

**Figure 4:**
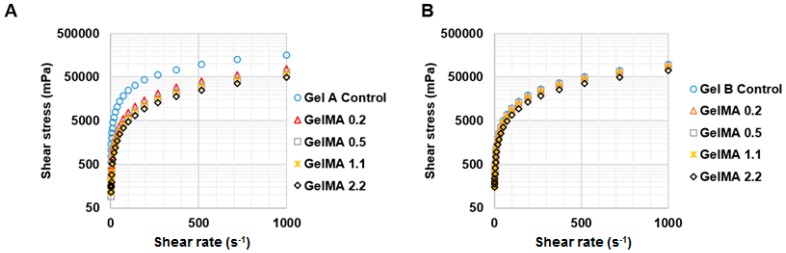

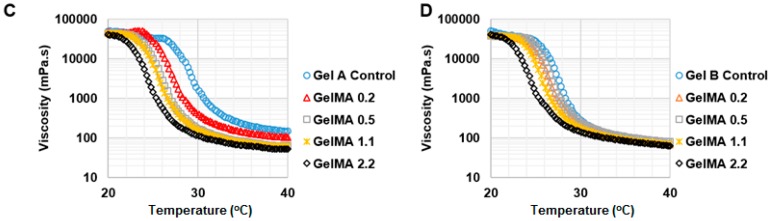
Rheological properties of types A and B GelMA. (**A**) Shear stress profile of 30% *w*/*v* type A GelMA at 37 °C (*n* = 3); (**B**) Shear stress profile of 30% *w*/*v* type B GelMA at 37 °C (*n* = 3); (**C**) Viscosity profile of 30% *w*/*v* type A GelMA as a function of temperature (*n* = 3); (**D**) Viscosity profile of 30% *w*/*v* type B GelMA as a function of temperature (*n* = 3).

We investigated the effect of shear rate on the shear stress and viscosity of types A and B GelMA in order to predict which DS and concentration of GelMA can be extruded. Figure 4 shows the shear stress and temperature sweep viscosity profiles of 30% *w*/*v* GelMA from type A and type B. The GelMA solutions at 37 °C from both types A and B behaved like shear thinning fluids. This can be seen as the viscosity of both types GelMA, as indicated by the slope of stress versus shear rate, decreased with increasing the shear rate. In types A and B GelMA solutions, GelMA with a higher DS exhibited lower shear stress and lower viscosity. Comparing type A GelMA and type B GelMA, it was observed that the overall shear stress and viscosity of type A GelMA were lower than type B GelMA. In a temperature sweep testing from 40 °C to 20 °C, the viscosity of all the GelMA solutions at 30% started to increase abruptly below 30 °C as seen in Figure 4C,D. The viscosity of the original gelatin solution started to increase at higher temperatures than GelMA solutions. The onset temperature of the abrupt increase in viscosity lowered with increasing the DS of the GelMA. Type A GelMA samples showed a more distinct viscosity profile as a function of temperature as compared to type B GelMA samples.

**Figure 5:**
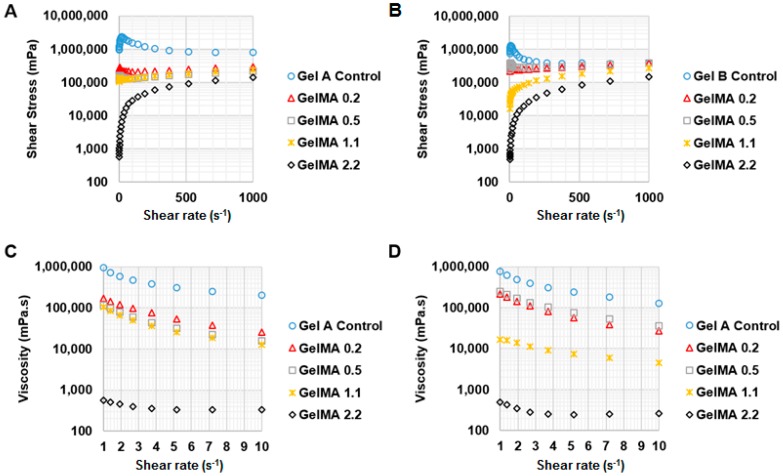
Shear thinning behaviour of types A and B GelMA at room temperature. (**A**) Overall shear stress profile of 20% *w*/*v* type A GelMA at 25 °C (*n* = 3); (**B**) Overall shear stress profile of 20% *w*/*v* type B GelMA at 25 °C (*n* = 3); (**C**) Viscosity profile of 20% *w*/*v* type A GelMA at 25 °C (*n* = 3); (**D**) Viscosity profile of 20% *w*/*v* type B GelMA at 25 °C (*n* = 3).

At 25 °C, GelMA at 30% *w*/*v* formed a physical gel and no longer exhibited shear thinning behaviour. Thus, we tested types A and B GelMA with 20% *w*/*v* on stress versus shear rate at 25 °C. The results are presented in Figure 5. Type A GelMA 2.2, type B GelMA 2.2 and type B GelMA 1.1 exhibited comparable pseudoplastic behaviours across the shear rate (1–1000 s^−1^) as the viscosity reduced with increasing the shear rate. Types A and B GelMA 2.2 solutions were selected for further investigation for 3D printing.

**Figure 6:**
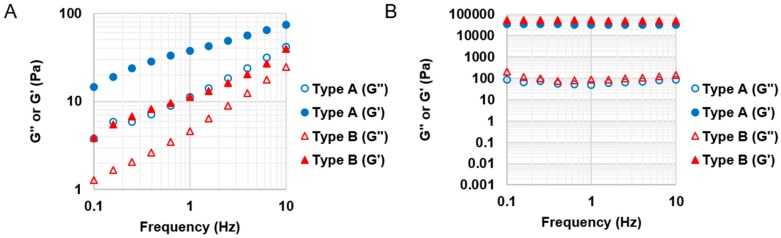
Viscoelastic properties of GelMA before and after curing. (**A**) Storage (G′) and loss modulus (G′′) of 20% *w*/*v* type A GelMA 2.2 and type B GelMA at 25 °C (*n* = 3). G′ values were higher than G′′ values across the frequency range for both of types A and B GelMA 2.2, which could provide initial shape fidelity after extrusion; (**B**) Storage (G′) and loss modulus (G′′) of cured 20% *w*/*v* types A and B GelMA 2.2 at 37 °C (*n* = 3). After curing, both types A and B GelMA 2.2 could show structural stability for G′ values are much higher than G′′ values across the frequency range.

A frequency sweep test was conducted to investigate the viscoelasticity of types A and B GelMA 2.2 solutions at 20% *w*/*v* at 25 °C as seen in Figure 6. Uncured types A and B GelMA 2.2 solutions exhibited a higher value of storage modulus than that of loss modulus at 25 °C. The storage modulus of type A GelMA 2.2 was higher than that of type B GelMA 2.2 across the frequency range of 0.1–1 Hz. After exposure to UV for 3 min, cured types A and B GelMA 2.2 samples showed more stability and higher storage moduli than uncured types A and B GelMA 2.2. After curing, both types A and B GelMA 2.2 showed structural stability as the G′ values were much higher than the G′′ values across the frequency range. Type B GelMA 2.2 exhibited a higher storage modulus than type A GelMA 2.2; the storage modulus of type B GelMA 2.2 is 53.1 ± 0.7 kPa, whereas that of type A GelMA 2.2 is 34.7 ± 2.4 kPa.

### 3.4. Printability of Types A and B GelMA

**Figure 7:**
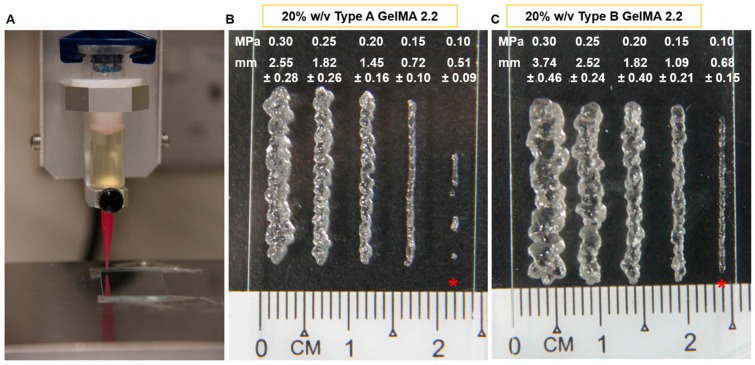
The effect of extrusion pressure on the printed line to find an optimal pressure for producing the best printed line. (**A**) Demonstration of the 3D extrusion style printing; (**B**) Type A GelMA 2.2 at 20% *w*/*v*; (**C**) Type B GelMA 2.2 at 20% *w*/*v*. * discontinuous lines.

Based on the rheology profiles obtained from the rheology testing, suitable samples (20% *w*/*v* types A and B GelMA 2.2) were selected for 3D extrusion style printing owing to their shear thinning behaviours, good shape fidelity at room temperature, and strong structural stability on curing. A trial was conducted to ensure that a proper fine line can be drawn during printing. Figure 7 showed that 0.10 MPa pressure produced discontinuous lines for both types A and B GelMA 2.2. The robot produced continuous lines of GelMA 2.2 above 0.15 MPa. However, the lines of type A GelMA were thinner than those of type B GelMA 2.2. Thus, 0.15 MPa was selected as the pressure for type A GelMA 2.2 and type B GelMA 2.2 extrusion at 20% *w*/*v*.

**Figure 8:**
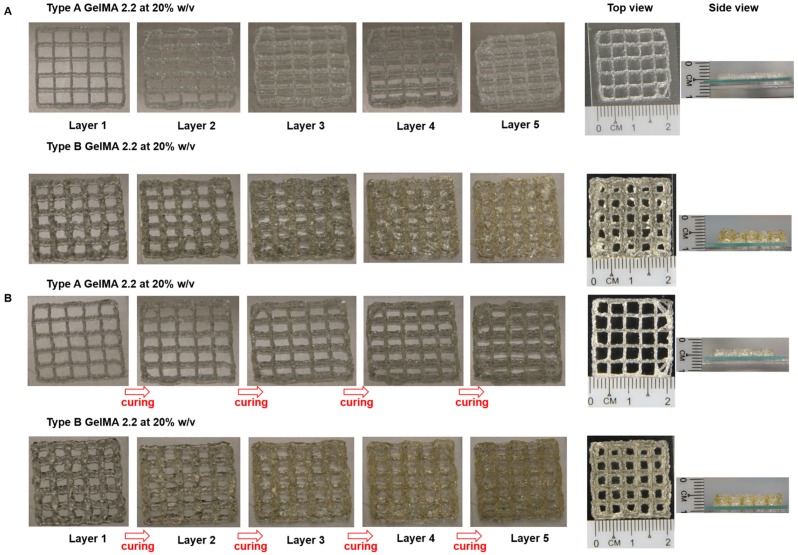
3D printing of 20% *w*/*v* GelMA containing 0.1% I2959 with five layers. (**A**) Types A and B GelMA 2.2 without curing; (**B**) Types A and B GelMA 2.2 with layer-by-layer curing by 365 nm U.V.

For the actual printing, a 3D grid pattern was designed in Microsoft Excel with the dimension of 2 cm × 2 cm with 4 mm line spacing. The 3D printing of 20% *w*/*v* type A GelMA 2.2 was compared between an uncured construct and a cured construct. Each of them was printed in five layers. Figure 8 presented the printing results of uncured and cured 20% *w*/*v* types A and B GelMA 2.2. The uncured constructs of types A and B GelMA displayed a defined grid pattern similar to the cured constructs of types A and B GelMA, respectively, as seen from the top view. In addition, type A GelMA produced a thinner construct compared to type B GelMA 2.2 as the extruded line (0.72 ± 0.10 mm) from type A GelMA was thinner from that (1.09 ± 0.21 mm) from type A GelMA under the same conditions.

### 3.5. 3D Printing of Cell-Laden Constructs of Types A and B GelMA

**Figure 9:**
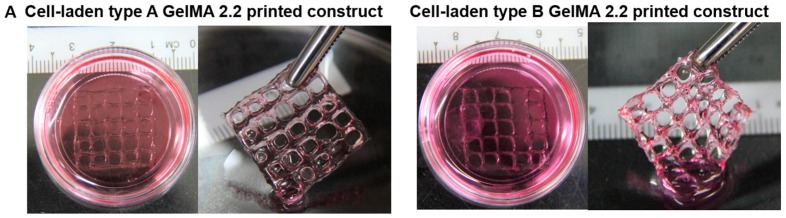

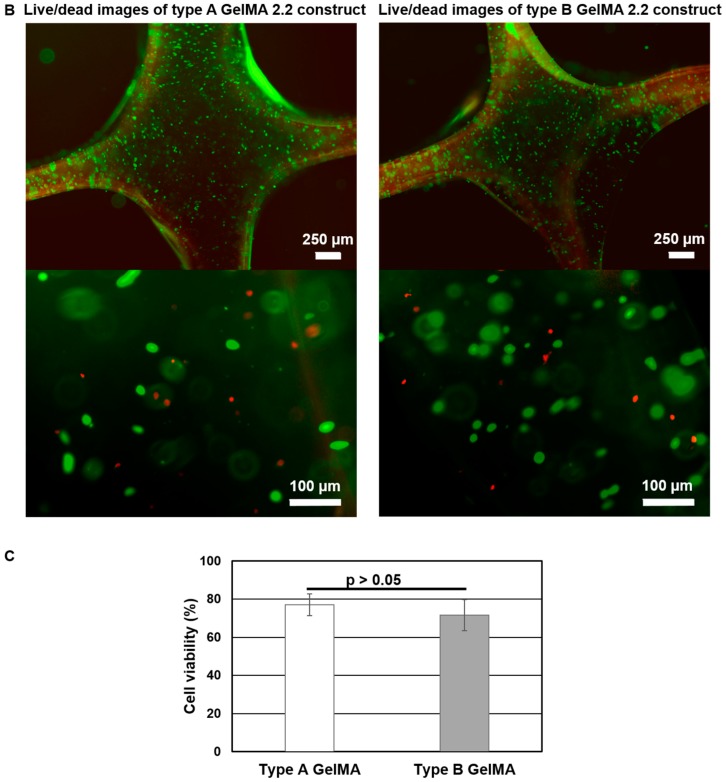
3D printing of Huh-7.5 cell-laden constructs of types A and B GelMA. (**A**) Gross pictures of cell-laden constructs of types A and B GelMA; (**B**) Live and dead staining images of cells in the cell-laden constructs of types A and B GelMA (Top: 2×; Bottom: 10×); (**C**) The viability of cells in the cell-laden constructs of types A and B GelMA.

In order to evaluate the cell viability of cell-laden printed constructs of types A an B GelMA, the live/dead assay was conducted using calcein-AM (green fluorescent staining) and ethidium homodimer-1 (red fluorescent staining). Huh-7.5 cell-laden printed constructs with five layers from types A and B GelMA at 20% containing 0.1% Irgacure were photocrosslinked at 1–2 mW·cm^−2^ of 365 nm for 5 min. Both types A and B GelMA printed constructs exhibited good shape fidelity and maintained their form when handled with a pair of forceps as seen in Figure 9A. Live/dead staining images showed that Huh-7.5 cells in the 3D printed constructs of types A and B GelMA were considerably viable (around 75%) and their viability was not significantly different as presented in Figure 9B.

## 4. Discussion

So far, most of the GelMA studies have focused on one type of GelMA, either type A or type B for their specific applications [16]. In order to compare type A GelMA with type B GelMA, type A and type B GelMA with a wide range of DS (15%–95%) were synthesized in a well controllable manner by the sequential MAA addition method using various feed ratios of MAA (mL) to gelatin (g) (0.1/1–0.0125/1). Type B GelMA exhibited higher degrees of substitution across the entire range as compared to type A GelMA, except at the highest feed ratio where the degree of substitution is close to the maximum. This may be due to the difference in IEP (isoelectric point) of type A and type B gelatin. Type B gelatin has a lower IEP of pH 4–6, compared to an IEP of pH 7–9 for type A gelatin. As the reaction of gelatin with MAA was maintained at pH 9, type B gelatin had more free amine group exposed owing to its lower IEP in the same reaction buffer. A larger number of freely available amino groups exposed in type B gelatin would be able to react with MAA and hence would lead to a higher degree of methacryloylation as compared to type A gelatin.

Both types A and B GelMA solutions were crosslinked within a few minutes to maintain their stable storage moduli after the exposure to a long wavelength UV (365 nm). The storage moduli of both types GelMA hydrogels were constant across the range of frequencies. The GelMA hydrogels also showed very good reproducibility, with all the storage moduli maintaining a relatively narrow standard deviation. The GelMA hydrogels with a higher storage modulus are more rigid and are able to withstand higher deformation stresses. Additionally, because the storage modulus is constant across the range of frequencies, the GelMA hydrogels are expected to maintain their elastic properties for a longer time period. The stiffness of GelMA hydrogels depends on their degrees of substitution (DS) and concentrations. A higher DS and concentration of GelMA solutions gave rise to hydrogels that have a higher storage modulus owing to the increased availability of crosslinkable methacryloyl groups (higher crosslinking density). Type B GelMA hydrogels exhibited a higher storage modulus than type A GelMA hydrogels possibly on the account of a slightly higher DS and a higher bloom number: Type A gelatin has a gel strength of 175 g bloom, whereas type B gelatin has a gel strength of 225 g bloom. Our GelMA hydrogels with various DS exhibit a wide range of stiffness (from 0.1 kPa to 150 kPa) which can be advantageous for diverse bioink applications. In situ photocrosslinkable properties of GelMA can secure a prolonged cell culture as well as structural stability of its scaffold.

The viscosity of GelMA solutions was varied as a function of temperature. GelMA with a higher DS tended to form a physical gel at lower temperatures. Gelatin formed a physical gel at higher temperature than GelMA. This could be due to the effect of the methacryloylation of GelMA, which may hinder the formation of physical bonds and helix structures in GelMA [15]. This phenomenon appeared to be more pronounced in type A GelMA than type B GelMA. Type A gelatin is less denatured and hydrolysed than type B gelatin in terms of IEP and the composition of amino acids [26]. Type A gelatin has more similarity of amino acid composition and IEP to collagen than type B gelatin. Therefore, the effect of the methacryloylation of type A gelatin on temperature-driven physical gelation was possibly more significant and obvious than that of type B GelMA. The viscosity of GelMA solutions decreased with increasing the shear rate at 37 °C, showing that GelMA solutions are pseudo-plastic (shear thinning) at 37 °C. However, GelMA at 30% *w*/*v* formed a strong physical gel at 25 °C, which rendered it unsuitable for extrusion-based 3D printing at room temperature, whereas type A and type B GelMA 2.2 at 20% *w*/*v* showed shear thinning properties under shear strain at 25 °C and extrusion through a syringe was possible.

Based on the material property studies, we chose 20% *w*/*v* type A GelMA 2.2 and type B GelMA 2.2 for further optimization of bioprinting parameters. Both types GelMA met two of the requirements of a bio-ink material which are shear thinning properties and shape fidelity. At the shear stress versus shear rate test, both types A and B GelMA solutions showed shear thinning behaviours at 25 °C as their viscosity reduced with increasing the shear rate. For shape fidelity, both types of GelMA exhibited higher storage moduli (G′) than their loss moduli (G′′) at 25 °C, which would probably allow the GelMA ink solutions to retain their shapes after printing. As the storage modulus of type A GelMA was higher than that of type B GelMA 2.2 at room temperature, the 3D printing construct of type A GelMA had a better resolution than that of type B GelMA under the same conditions. Compared to type B gelatin, type A gelatin is more similar to the original collagen in terms of amino acid components, IEP and capability of helix structure formation. In this respect, type A GelMA can maintain better physical gelation properties at room temperature than type B GelMA, leading to good shape fidelity. Both types A and B GelMA printing constructs presented structural stability after layer-by-layer curing.

Cell-laden printed constructs of types A and B GelMA at 20% showed good shape fidelity in culture media and could be easily handled with a pair of forceps. Both of them exhibited good viability (around 75%). In summary, we synthesized both types of GelMA, compared their viscoelastic properties, and investigated their feasibility as bioinks for 3D printing. Even though increasing the concentration of bioinks to obtain shape fidelity is a common strategy, the high concentration of a printing ink may compromise cell viability during cell culture. As the printability of GelMA is considerably sensitive to the temperature change, a lower concentration of GelMA can be used for 3D printing once 3D printers are equipped with a more precise temperature control system from low (5 °C) to high (37 °C). In addition, the employment of viscosity enhancers such as Ca^++^, Mg^++^, and other small molecules could reduce the concentration of GelMA for 3D printing, and that of various bioactive molecules could provide GelMA with more attractive features as bioink materials [29].

## 5. Conclusions

The aim of this work was to synthesize GelMA from different types of gelatin precursors (type A and type B) and compare them in terms of degree of substitution, mechanical properties, and feasibility for bioink applications. Both types of gelatin reacted with MAA to form gelatin methacryloyl (GelMA) in a similar pattern; the degree of methacryloylation of type B GelMA was slightly higher than that of type A GelMA at the same feed ratio of MAA to gelatin. In terms of mechanical properties, an increase in storage modulus could be brought about by increasing the degree of substitution of GelMA, GelMA concentration, and by using GelMA with higher bloom strength. For printability of GelMA as a bioink, both types A and B GelMA solutions with the high DS exhibited shear thinning properties, shape fidelity at 25 °C, and structural stability on curing. Type A GelMA produced the printed construct with a better resolution that type B GelMA at the same extrusion pressure. Cell-laden GelMA printed constructs had an easy-to-handle feature in culture media. Huh-7 cells in both types A and B GelMA printed constructs displayed relatively high viability even at 20% concentration.

## Supplementary Materials

The following are available online at www.mdpi.com/1996-1944/9/10/797/s1, Table S1: Comparison of type A and type B GelMA in terms of DS and storage modulus, Figure S1: Reaction of types A and B gelatin with MAA via sequential MMA addition, Figure S2: Viscosity versus temperature profile in type A GelMA of 30%, 20%, and 10% *w*/*v*, Figure S3: Double logarithmic flow Curve, Figure S4: Flow behaviour of 20% type A and type B GelMA 2.2, Figure S5: Flow behaviour index of 20% type A and type B GelMA 2.2, Figure S6: Flow rate profile versus applied pressure, and Figure S7: Shear rate profile versus applied pressure, Equation S1: Apparent viscosity in power law function, Equation S2: Shear rate of non-Newtonian fluid under conical cylindrical geometry.
