# Supplementary material for: Synthesis and Characterization of Types A and B Gelatin Methacryloyl for Bioink Applications
Source: Materials (Basel). 2016 Sep 24;9(10):797. doi: 10.3390/ma9100797 (PMC5456596; doi:10.3390/ma9100797)
Supplement: Supplementary file 1 [file materials-09-00797-s001.pdf]

Supplementary Materials: Synthesis and Characterization of Types A and B Gelatin Methacryloyl for Bioink Applications

Bae Hoon Lee, Nathaniel Lum, Li Yuan Seow, Pei Qi Lim and Lay Poh Tan

Table S1. Comparison of type A and type B GelMA in terms of DS and storage modulus.

Feed Ratio (MAA (mL)/Gelatin (g))	Gelatin Type	GelMA	DS (%)	Storage Modulus (kPa, at 30%)	Storage Modulus (kPa, at 20%)	Storage Modulus (kPa, at 10%)
0.1/1	A	GelMA 2.2	94.9 ± 0.5	67.6 ± 4.8	34.7 ± 2.4	9.1 ± 0.4
	B	GelMA 2.2	94.9 ± 0.6	147.3 ± 11.4	53.1 ± 0.7	10.0 ± 0.7
0.05/1	A	GelMA 1.1	72.6 ± 0.6	26.7 ± 0.8	19.5 ± 0.9	5.1 ± 0.2
	B	GelMA 1.1	82.7 ± 1.5	78.1 ± 2.8	28.1 ± 2.6	6.3 ± 0.4
0.025/1	A	GelMA 0.5	39.5 ± 1.8	11.3 ± 0.2	5.1 ± 0.8	1.5 ± 0.1
	B	GelMA 0.5	45.7 ± 3.5	25.4 ± 0.7	9.7 ± 0.1	2.3 ± 0.1
0.0125/1	A	GelMA 0.2	14.8 ± 0.8	1.9 ± 0.1	1.1 ± 0.1	0.1 ± 0.1
	B	GelMA 0.2	20.7 ± 6.4	5.3 ± 0.3	2.1 ± 0.1	0.3 ± 0.1

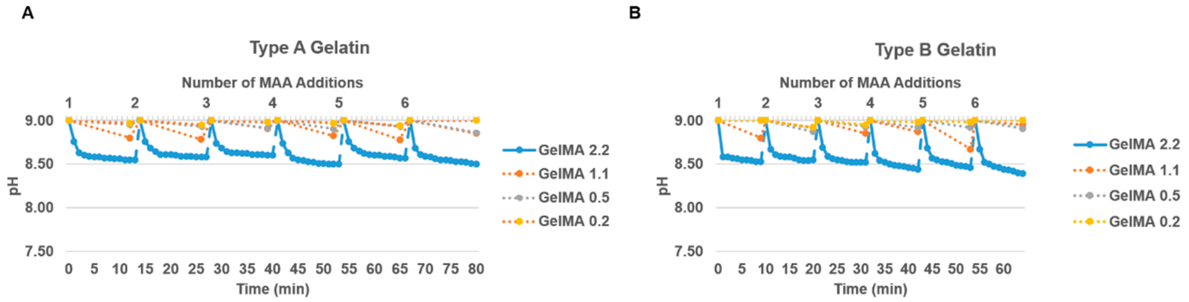

Figure S1. Reaction of types A and B gelatin with MAA via sequential MMA addition. (**A**) pH change versus time plot for type A gelatin; (**B**) pH change versus time plot for type B gelatin. The pH change versus time profile in types A and B gelatin showed a similar pattern. The immediate decrease in pH following addition of MAA within a few minutes was the result of the available MAA being used up in the reaction and consequently being unable to contribute to maintaining pH 9 owing to the generation of MA (methacrylic acid). This indicated that the reaction of MAA with both types of gelatin occurred quickly upon collision. The magnitude of the decrease in pH was more pronounced for GelMA with higher feed ratios owing to the production of more MA.

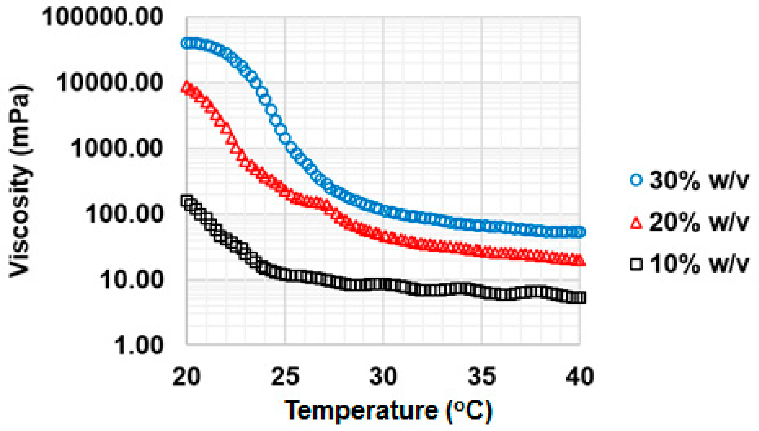

Figure S2. Viscosity versus temperature profile in type A GelMA of 30%, 20%, and 10% *w*/*v* (n = 3). The viscosity of type A GelMA increased with decreasing the temperature. The viscosity of GelMA was also in proportion to the concentration of GelMA. GelMA at 30% formed the most stable and strongest physical gel at room temperature among the samples.

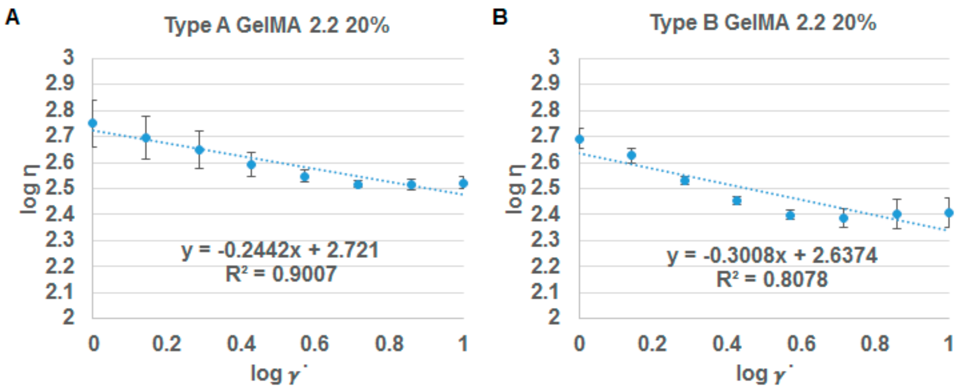

Figure S3. Double logarithmic flow curve. (**A**) Type A GelMA 2.2 20% (n = 3); (**B**) Type B GelMA 2.2 20% (n = 3).

Equation S1. Apparent viscosity in power law function [1].

$$\eta = \frac{\tau}{\dot{\gamma}} = k(\dot{\gamma})^{n-1}$$

$$\log \eta = \log k + (n-1) \log \dot{\gamma}$$

where,

$$\eta = Viscosity$$

$$\tau = Shear\ stress$$

$$\dot{\gamma} = Shear\ rate$$

$$k = Fluid\ consistency\ coefficient$$

$$n = Flow\ behaviour\ index,$$

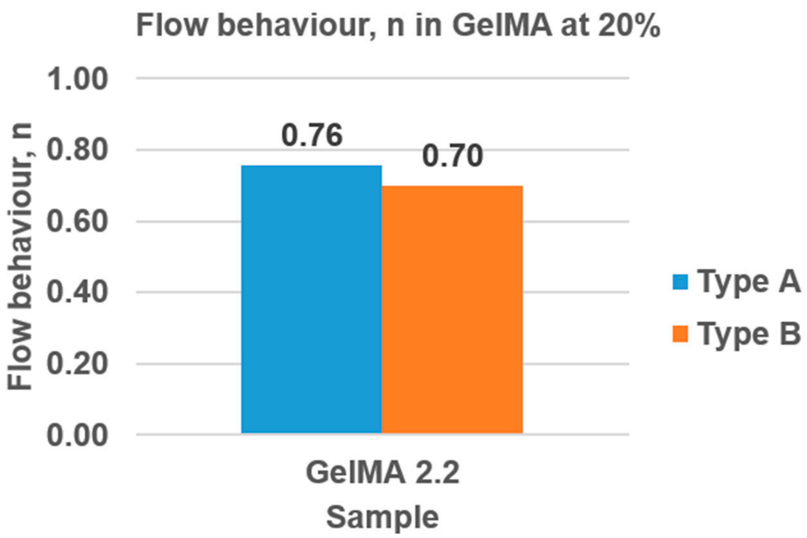

Figure S4. Flow behaviour index of 20% type A and type B GelMA 2.2; both types of GelMA solutions behaved like pseudoplastic (shear thinning) fluids for n is less than 1.

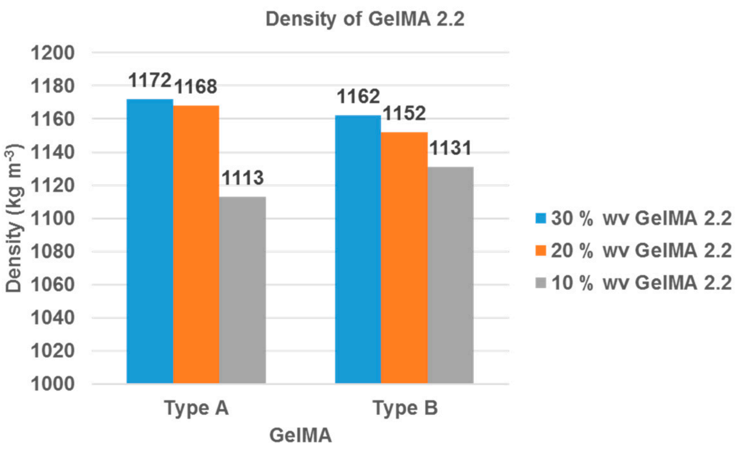

Figure S5. Density estimation of type A and type B GelMA 2.2 based on weighing 100 μL of GelMA 2.2 samples.

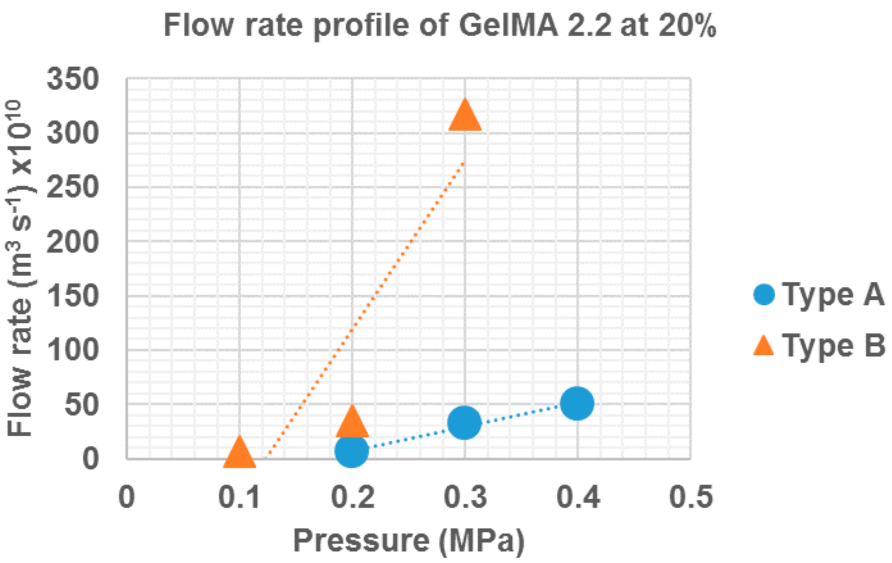

Figure S6. Flow rate versus applied pressure profile of 20% type A and type B GelMA 2.2.

Equation S2. Shear rate of non-Newtonian fluid under conical cylindrical geometry [2].

$$\dot{\gamma} = \left(\frac{3n+1}{n}\right)\frac{Q}{\pi r^3}$$

where,

$$\dot{\gamma} = Shear\ rate$$

$$n = Flow\ behaviour\ index$$

$$Q\ = Flow\ rate$$

$$r = Radius$$

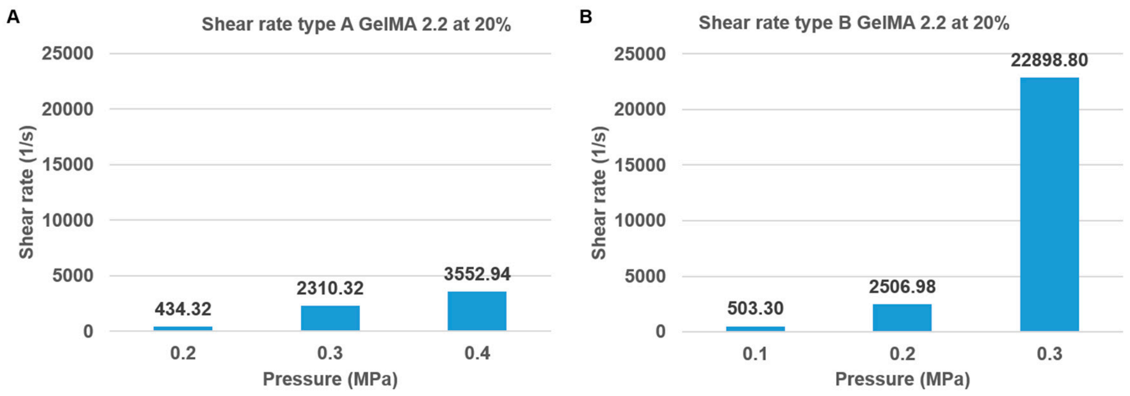

Figure S7. Shear rate versus applied pressure profile of 20% type A and type B GelMA 2.2 based on Equation S2.

References

1. Chhabra, R.P.; Richardson, J.F. Non-Newtonian Fluid Behaviour. In *Non-Newtonian Flow and Applied Rheology*, 2nd ed.; Chhabra, R.P., Richardson, J.F., Eds.; Butterworth-Heinemann: Oxford, UK, 2008; Chapter 1, pp. 1–55.
2. Crawford, R.J. Analysis of Polymer Melt Flow. In *Plastics Engineering*, 2nd ed.; Elsevier: Amsterdam, The Netherlands, 2013; Chapter 5.
